# Tibial Insufficiency Fracture with Characteristics of an Atypical Fracture: A Rare Case and Literature Review

**DOI:** 10.3390/medicina60111814

**Published:** 2024-11-04

**Authors:** Ju-Yeong Kim, Se-Won Lee

**Affiliations:** 1 Department of Orthopedic Surgery, Gyeongsang National University Changwon Hospital, Gyeongsang National University School of Medicine, 11 Samjeongja-ro, Seongsan-gu, Changwon 51472, Republic of Korea; passion0309@hanmail.net; 2 Department of Orthopedic Surgery, Yeouido St. Mary’s Hospital, College of Medicine, The Catholic University of Korea, 10, 63-Ro, Yeongdeungpo-Gu, Seoul 07345, Republic of Korea

**Keywords:** tibia, atypical insufficiency fracture, bisphosphonate, osteoporosis

## Abstract

*Background and Objectives*: Osteoporosis is becoming more prevalent with the rise in the aging population, leading to the increased use of bisphosphonates for treatment. While these medications are effective in preventing osteoporotic fractures, long-term use has been associated with atypical insufficiency fractures, primarily in the femur. However, atypical fractures in other weight-bearing bones, such as the tibia, have rarely been reported. This study aims to present a case of an atypical insufficiency fracture of the tibia in an elderly female who has been on long-term bisphosphonate therapy and to review treatment outcomes within the context of the current literature. *Patient concerns*: A 76-year-old female presented with pain in the proximal right tibia, developing two months prior without trauma. She had been receiving long-term bisphosphonate therapy for osteoporosis, initially taking sodium risedronate orally for 4 years, followed by intravenous ibandronate for 10 years. Physical examination revealed localized tenderness, and radiographs showed cortical thickening and a horizontal fracture line in the proximal right tibia. MRI confirmed these findings, along with surrounding edema. The laboratory results were mostly normal, but the bone formation marker osteocalcin was significantly reduced. The patient had a history of insufficiency fractures in the ipsilateral tibia and contralateral femur, previously treated conservatively with teriparatide. A similar conservative approach was attempted but failed, leading to surgical intervention with intramedullary nailing and supplementary plating. At the 8-month follow-up, the patient showed successful fracture union and resolution of symptoms. *Conclusion:* Long-term use of bisphosphonates, though effective for osteoporosis, can lead to atypical insufficiency fractures, primarily in the femur but also occasionally in the tibia. Clinicians should consider this possibility when patients present with pain in weight-bearing bones without a history of trauma. Prompt diagnosis through thorough history-taking, physical examination, and appropriate imaging is essential to ensure timely management.

## 1. Introduction

Osteoporosis is increasing as a result of longer life spans and an aging population [1]. The use of bisphosphonate drugs has been increasing for the treatment of osteoporosis. These drugs are generally regarded as relatively safe [2]. An association between long-term use of the anti-bone resorptive agent bisphosphonate and atypical insufficiency fractures of the femur has been well documented [3]. In a recent large cohort study, it was found that patients on bisphosphonates for over 8 years were more than 40 times more likely to develop atypical femoral fractures compared with patients with less than 1 year of usage [4]. It has been suggested that the higher incidence of atypical femoral fractures in patients with high adherence to bisphosphonate therapy indicates that this drug may play a significant role in the pathogenesis of these fractures [5]. Fractures in other long bones such as the tibia have only rarely been reported [6,7]. As a result, in clinical settings, the diagnosis of atypical tibial fractures is frequently delayed, leading to suboptimal treatment outcomes. Recognizing that atypical fractures can also be present in the tibia underscores the need for discussions on precise diagnostic strategies and effective treatment approaches. This paper aims to provide clinically relevant insights and contributes valuable knowledge to the field.

The authors report a case of an atypical insufficiency fracture of the tibia without trauma in a 76-year-old female patient who had been on long-term bisphosphonate therapy. This case is presented along with a review of the literature regarding the treatment outcomes. Notably, unlike previous cases, this case is distinguished by the occurrence of multiple atypical fractures at various sites in a single patient who had been on long-term bisphosphonate therapy, highlighting a distinctive clinical presentation.

## 2. Case Presentation

**Figure 1:**
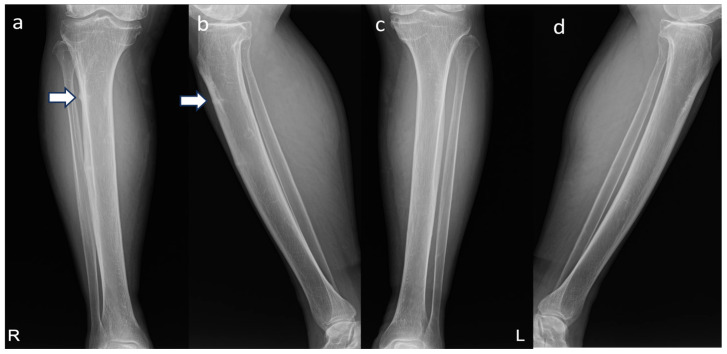
Preoperative radiographs of both tibiae. (**a**) Anteroposterior (AP) view of the right tibia showing cortical thickening and a short, horizontal radiolucent fracture line in the anterolateral cortex of the proximal tibia (white arrows). (**b**) Lateral view of the right tibia demonstrating the same findings. (**c**) AP view and (**d**) lateral view of the left tibia showing no abnormal findings.

A 76-year-old female patient presented with pain in the proximal right tibia, which had developed two months prior without any history of trauma. The patient had been taking the bisphosphonate sodium risedronate (Actonel^®^; Sanofi-Aventis, Paris, France) 150 mg orally once a month for 4 years for osteoporosis, followed by intravenous administration of the bisphosphonate ibandronate sodium monohydrate (Bonviva^®^; Roche, Basel, Switzerland) 3 mg every three months for the subsequent 10 years. On physical examination, localized tenderness was observed in the anterior aspect of the proximal right tibia. Plain anteroposterior radiographs showed cortical thickening and a short, horizontal radiolucent fracture line in the anterolateral cortex of the proximal right tibia (Figure 1), with no abnormal findings observed in the radiographs of the contralateral side.

**Figure 2:**
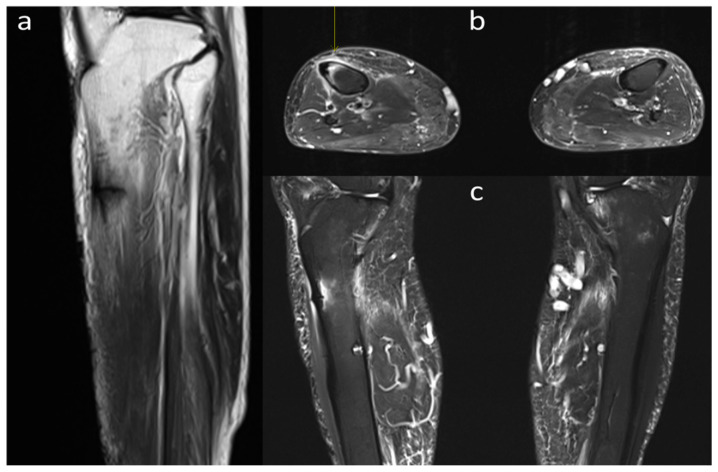
Preoperative MRI of the tibiae. (**a**) T1-weighted sagittal image of the right tibia showing cortical thickening and a horizontal fracture line in the anterolateral aspect of the mid-shaft. (**b**) T2-weighted axial image and (**c**) T2-weighted coronal image of both tibiae.

MRI also revealed cortical thickening in the anterolateral aspect of the left mid-shaft tibia, along with a horizontal fracture line. Edema was observed around the insufficiency fracture line (Figure 2).

**Figure 3:**
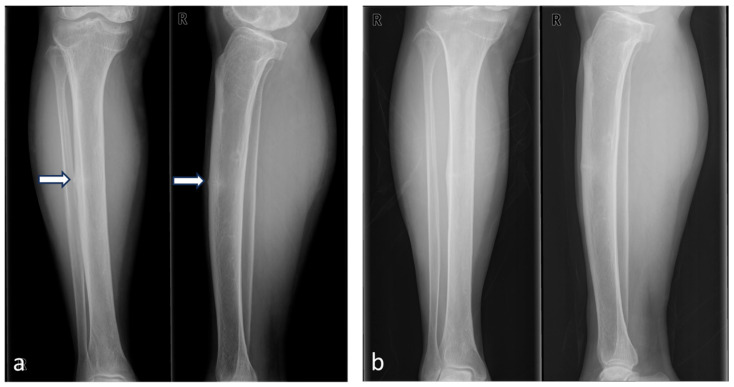
Comparative radiographs of the right tibia demonstrating the course of an atypical fracture. (**a**) The left images, taken 17 years before the patient’s 2023 presentation (in 2006), show a fatigue fracture in the mid-shaft of the right tibia (white arrow). At this time, surgical fixation was recommended but declined by the patient. (**b**) The subsequent images, taken three years later in 2009, reveal complete union of the fracture.

**Figure 4:**
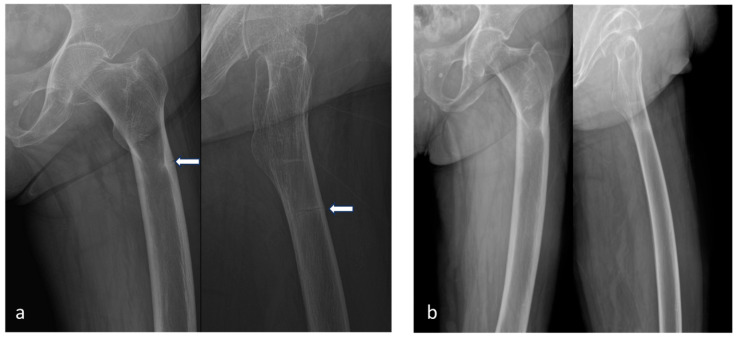
Comparative radiographs of the left femur demonstrating the course of an atypical fracture. (**a**) The left images, taken 6 years before the patient’s 2023 presentation (in 2017), show a fatigue fracture in the lateral cortex of the left femoral shaft (white arrow). (**b**) The subsequent images, taken one year later in 2018, reveal complete union of the fracture, demonstrating spontaneous healing without surgical intervention.

Blood tests revealed normal levels of parathyroid hormone, thyroid function tests, serum calcium, serum phosphate, alkaline phosphatase, and 25-hydroxy vitamin D. However, the bone formation marker osteocalcin was decreased at 4.82 ng/mL (reference range: 11–30 ng/mL). The patient had a notable medical history of previous insufficiency fractures in the ipsilateral tibia (Figure 3) and contralateral femur (Figure 4).

**Figure 5:**
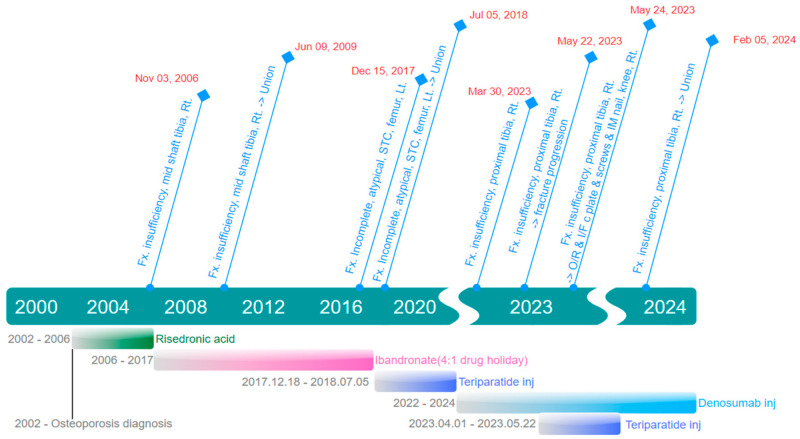
Timeline of the patient’s osteoporosis treatment history and the occurrence of atypical fractures from 2002 to 2024. The chart details the sequence of anti-osteoporotic medications prescribed and the timing of key fracture events over a 22-year period.

These fractures had been treated conservatively with teriparatide injections, resulting in fracture union and improvement in symptoms. Due to the patient’s previous success with conservative treatment, a similar conservative approach was taken this time, utilizing the parathyroid hormone analog (rhPTH 1–34), the bone-forming osteoporosis medication teriparatide (Forsteo^®^; Eli Lilly, Indianapolis, IN, USA), along with calcium and vitamin D supplements, and activity restriction. The timeline illustrates the initiation and discontinuation dates of osteoporosis medications. To provide additional details, bisphosphonate therapy was administered for 15 years from 2002 to 2017. After an atypical femoral fracture occurred in 2017, conservative treatment with teriparatide was maintained for 8 months until femoral union was confirmed, after which the therapy was transitioned to denosumab as maintenance therapy. Subsequently, an atypical fracture developed in the proximal tibia in 2023, and a short course of teriparatide was administered again. Since then, osteoporosis management has been maintained with continued denosumab therapy (Figure 5).

**Figure 6:**
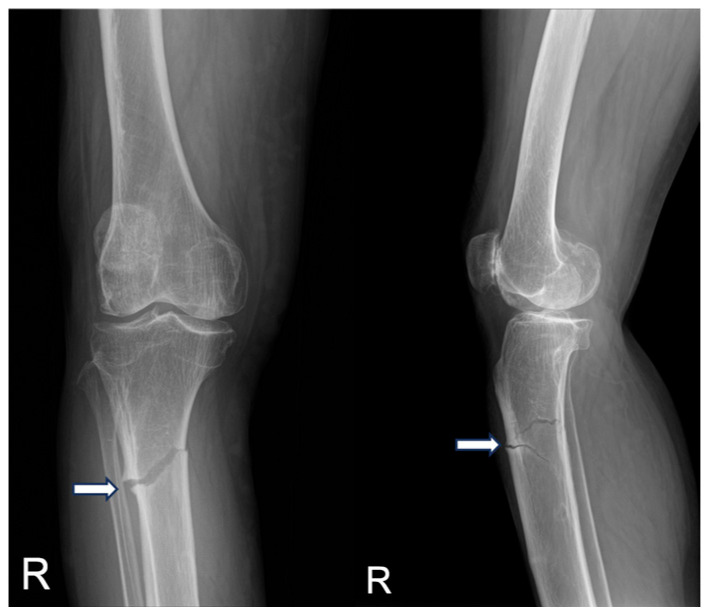
Follow-up radiograph of the right proximal tibia. The image demonstrates an extension of the fracture line (white arrows) compared to the initial presentation, indicating progression of the atypical fracture after the patient experienced sudden worsening of pain while exiting a car.

However, the patient’s symptoms did not improve, and pain persisted, which suddenly worsened while stepping out of a car, leading to her return to the hospital. Follow-up plain anteroposterior radiographs showed extension of the fracture line in the proximal right tibia compared to the previous images (Figure 6).

**Figure 7:**
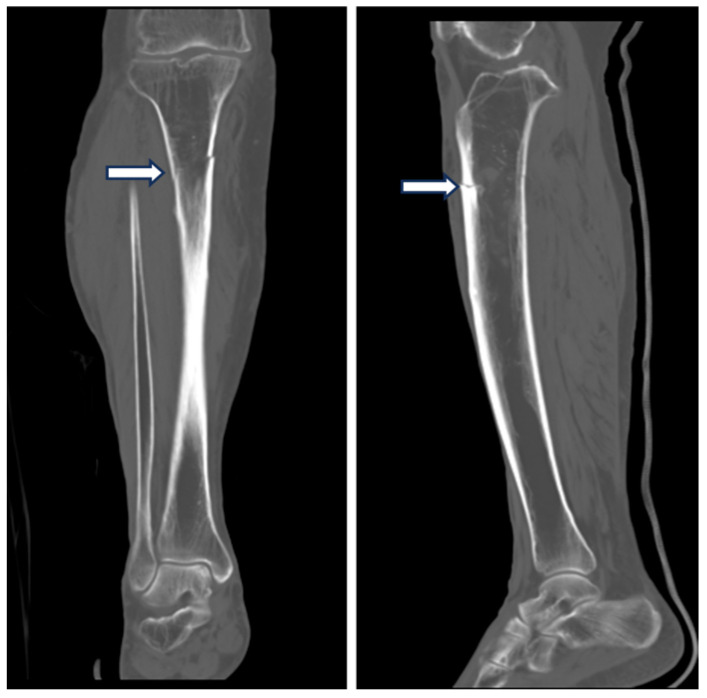
Computed tomography (CT) images of the proximal right tibia. The scans demonstrate cortical thickening in the anterolateral aspect, accompanied by a horizontal fracture line (white arrows), providing a more detailed view of the atypical fracture progression.

Computed tomography also revealed cortical thickening in the anterolateral aspect of the proximal right tibia, along with a horizontal fracture line (Figure 7).

**Figure 8:**
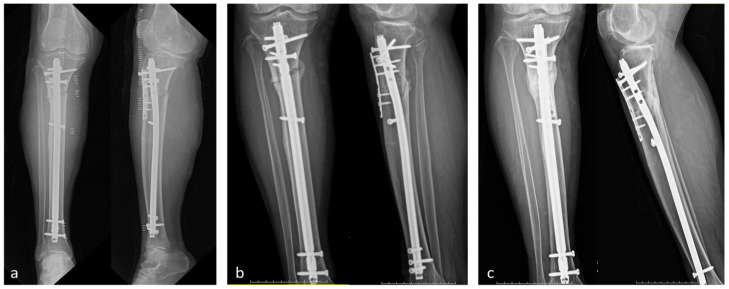
Sequential radiographs demonstrating the progression of fracture healing following intramedullary nailing and supplementary plating of the right proximal tibia. (**a**) Immediate postoperative radiograph showing the Tibial Nail and 1/3 Tubular Plate in place. (**b**) Three-month follow-up radiograph revealing initial signs of fracture healing. (**c**) Eight-month follow-up radiograph demonstrating significant progression of fracture union with callus formation.

Intramedullary nailing using a Tibial Nail (Synthes^®^, Oberdorf, Switzerland) with supplementary plating using a 1/3 Tubular Plate (Synthes^®^, Oberdorf, Switzerland) was performed to address the lesion. The patient showed a gradual reduction in pain postoperatively, with complete resolution of pain at approximately 3 months after surgery. At the 8-month follow-up, radiographic evaluation demonstrated evidence of progressing fracture union (Figure 8).

## 3. Discussion

Osteoporosis is increasing as a result of longer life spans and an aging population. The use of bisphosphonate drugs has been increasing for the treatment of osteoporosis [1,2]. Bisphosphonates are known to increase bone mass by inhibiting osteoclast activity, thereby reducing bone turnover [5]. Bisphosphonates have been used as first-line medications for the prevention of osteoporotic fractures; however, long-term use has been associated with excessive suppression of bone remodeling, which impairs the mechanical strength of the bone and delays the repair of microdamage, thereby increasing the risk of atypical non-traumatic fractures, particularly in the femur [3]. While various cases of atypical insufficiency fractures in the subtrochanteric and diaphyseal regions of the femur have been reported in patients with long-term bisphosphonate use, atypical insufficiency fractures in long bones outside the femur are relatively rare [6,7]. Stress fractures can be broadly categorized into fatigue fractures and insufficiency fractures. Fatigue fractures occur when abnormal repetitive forces are applied to normal bone, whereas insufficiency fractures are defined as fractures that occur when normal forces are applied to abnormal bone [8,9]. Fatigue fractures most commonly occur in the tibia, particularly in young, physically active individuals such as military personnel or athletes, and are exceedingly rare in elderly patients. In contrast, insufficiency fractures primarily affect postmenopausal women, typically occurring without trauma, and are most frequently reported in bones such as the pelvis, sacrum, and femur, while being infrequently observed in the tibia [10]. Risk factors that increase the incidence of insufficiency fractures include osteoporosis, osteomalacia, hyperparathyroidism, rheumatoid arthritis, and diabetes. Additionally, long-term use of medications such as steroids, statins, proton pump inhibitors, and hormone replacement therapy, as well as prolonged bisphosphonate therapy, is known to further elevate the risk [3]. In this case, the patient sustained a fracture in the diaphysis of the tibia without any significant history of trauma. Initially, a fatigue fracture, which commonly occurs in the tibia without trauma, was considered. However, given the patient’s age, relatively low level of physical activity, and the location of the fracture on the anterolateral aspect of the tibia, an insufficiency fracture was deemed more likely than a fatigue fracture. There are no specific criteria for atypical fractures of the tibia; thus, this case was evaluated based on the 2013 criteria for atypical femoral fractures defined by the American Society for Bone and Mineral Research (ASBMR) [11]. The patient met four major features: the fracture occurred with minimal or no trauma, the fracture initiated at the lateral cortex and exhibited a transverse pattern, it was an incomplete fracture involving only the lateral cortex until progression to a complete fracture, and there was cortical thickening around the fracture site with periosteal or endosteal breaking resembling a ‘beak-like’ appearance. Additionally, the patient met three minor features: generalized cortical thickening, prodromal symptoms, and delayed fracture healing. Given these findings, this case is significant as it represents a rare instance of an atypical insufficiency fracture of the tibia in an elderly patient. We reviewed previously reported cases of atypical insufficiency fractures of the tibia (Table 1).

According to the other literature, Bissonnette et al. reported a case of an atypical insufficiency fracture of the tibial diaphysis in a middle-aged female patient who had been taking bisphosphonates for four years [6]. They highlighted the possibility of atypical insufficiency fractures occurring in long bones other than the femur, particularly in weight-bearing bones, in patients on bisphosphonate therapy. Furthermore, they emphasized the need for awareness of this risk and called for additional research on the topic. Odvina et al. reported a case of an atypical fracture of the right tibia in a postmenopausal middle-aged woman who had been taking bisphosphonates for three years [12]. Histological examination through bone biopsy revealed severe suppression of bone formation. Additionally, insufficiency fractures in long bones other than the tibia, associated with bisphosphonate use, have also been reported. Breglia and Carter reported a case of an insufficiency fracture of the tibia in a 43-year-old Caucasian woman who had been taking bisphosphonates, with improvement observed following conservative treatment [7]. Considering the treatment outcomes of previous cases, initial management often involved switching osteoporosis medications and opting for conservative treatment with non-weight-bearing measures. Surgical intervention was reserved for cases where conservative treatment failed or when the symptoms or fracture displacement were severe. It was also noted that the healing process was generally delayed on average. In this case, despite attempts at conservative treatment through a change in osteoporosis medication, bone union did not progress, and the fracture extended anteriorly, with persistent pain. Ultimately, surgical treatment with intramedullary nailing and plating was performed. Delayed bone union was achieved at 8 months, resulting in a successful outcome.

Unlike previous cases, this case is remarkable and distinctive in that a single patient who had been on long-term bisphosphonate therapy presented with multiple atypical fractures in various locations. Non-surgical treatment was initially chosen for each of the three fractures occurring in different locations. For the first atypical fracture in the right femur, bisphosphonate therapy was adjusted, resulting in successful union. For the second fracture in the left femur, bisphosphonate therapy was discontinued, and treatment with teriparatide and denosumab led to fracture union. This approach was based on studies supporting the effectiveness of switching to teriparatide and denosumab following the occurrence of atypical fractures after prolonged bisphosphonate use [18,19].

For the third fracture in the tibia, conservative treatment was initially attempted; however, successful union was ultimately achieved only through surgical intervention. The duration of bisphosphonate use in this case exceeded 10 years, indicating poor bone quality, as studies have shown that extended bisphosphonate therapy can reduce the fracture healing rate [20,21]. In our case and a review of the similar literature, delayed or non-union has frequently been observed in patients who used bisphosphonate therapy for over 10 years. This suggests that surgical treatment may be more beneficial than conservative management in such instances.

While non-surgical treatment was applied to each fracture, outcomes varied according to the location and timing of each fracture. This may imply that while all three fractures share a common underlying cause, the severity differed. The occurrence of three fractures in the same patient is likely related to prolonged bisphosphonate therapy lasting over 15 years, coupled with adverse effects from continued maintenance therapy with denosumab. The mechanisms of these medications may reduce bone quality and increase fragility, leading to atypical fractures in the commonly affected bilateral femur and, ultimately, the rarely reported tibia.

This study has limitations inherent to a simple case report, particularly the lack of analysis regarding factors other than bisphosphonates that may contribute to insufficiency fractures. Additionally, there were no baseline data on bone formation and resorption markers prior to the initiation of bisphosphonate therapy, making it impossible to determine the extent of suppression after its use. Additionally, the small sample size presents a limitation in standardizing treatment and prognosis. In summary, while most reported cases of atypical insufficiency fractures in patients on bisphosphonate therapy have involved the femur, there is a possibility for such fractures to also occur in long bones outside of the femur, particularly in weight-bearing areas. Therefore, in patients who have been on long-term bisphosphonate therapy and present with pain in weight-bearing long bones without any history of trauma, the possibility of atypical insufficiency fractures should be considered. Thorough history taking, physical examination, and plain radiographs are essential, and if fractures are not detected on radiographs, further diagnostic modalities such as MRI or bone scintigraphy should be considered. Moreover, further studies are needed to clarify the precise relationship between bisphosphonate therapy and atypical insufficiency fractures.

## 4. Conclusions

It is important to recognize the potential association between long-term bisphosphonate therapy, a commonly used treatment for osteoporosis, and atypical insufficiency fractures. Although these fractures most commonly occur in the femur, they can also occur in the tibia, albeit rarely. In clinical practice, when patients present with pain in weight-bearing long bones, the possibility of an atypical insufficiency fracture should be considered. A thorough patient history, physical examination, and plain radiographs, along with additional diagnostic tests, should be performed as necessary.

## Figures and Tables

**Table 1:** Current published case reports on atypical tibial fractures associated with long-term bisphosphonate/antiresorptive use.

Case Report	Age/Sex	Antiresorptive Agent (Duration)	Fracture Site	Treatment	Outcome
Odvina et al., 2010 [12] ^1^	38–77/F	Alendronate (10), Risedronate (3), 2–11 years	Femoral shaft (11), tibial (1), humeral (1)	Bone biopsy (6)	Delayed/non-healing
Imbuldeniya et al., 2012 [13]	76/F	Pamidronate (6 years, post-alendronate 3 years)	Proximal tibiae (bilateral), distal femur	Conservative	Full resolution in 2 months
Bissonnette et al., 2013 [6]	77/F	Zoledronic acid (4 years)	Left tibial diaphysis	IM nail fixation	Good healing at 4 months
Cecchetti et al., 2015 [14]	58/F	Risedronate (5 years)	L tibia, bilateral tibial plateau, L femoral/tibial metaphysis, R tibia, calcaneus, talus	Conservative, teriparatide	Recurrent fractures
Cecchetti et al., 2015 [14]	78/F	Alendronate (5 years)	Right tibial diaphysis	Conservative	Consolidation in 2 months
Schimpf et al., 2018 [15]	76/F	Risedronate (5 years), Strontium ranelate (2 years), Zoledronate (4 years), denosumab (2 years)	Bilateral distal tibial metaphysis	Conservative (L), surgery (R)	Delayed healing (L), healed (R)
Malabu et al., 2019 [16]	63/F	Alendronate (intermittent, 4 years)	Right proximal tibial shaft	Conservative	Successful healing
Suthar et al., 2021 [17]	65/F	Risedronate (5 years)	Left tibial plateau	Conservative	Full recovery at 8 months

^1^ The data from “Odvina et al., 2010 [12]” represent 13 female patients aged between 38 and 77 years. They were treated with alendronate (10 patients) or risedronate (3 patients) for 2 to 11 years. The main fracture sites included the femoral shaft (11 patients), tibia (1 patient), and humerus (1 patient). Bone biopsy was performed in 6 patients, and most cases exhibited delayed or non-healing fractures.

## Data Availability

All data concerning the case are presented in the manuscript.
